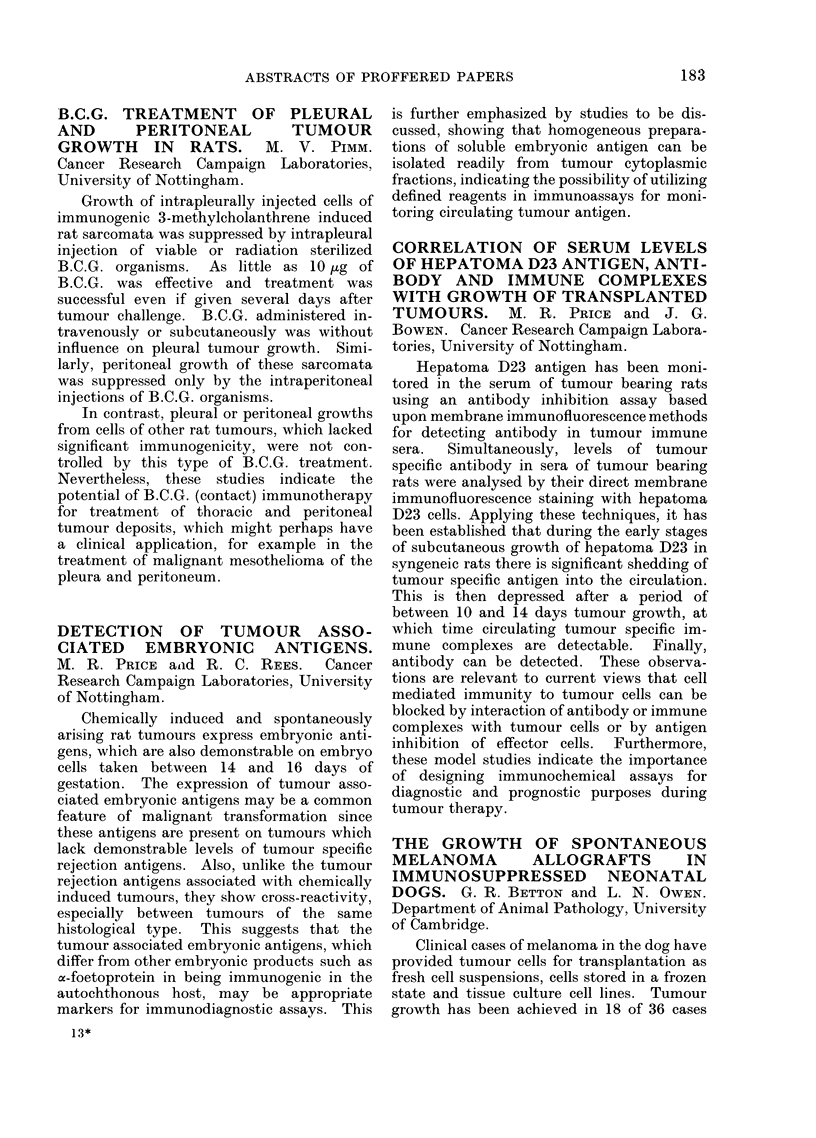# Proceedings: B.C.G. treatment of pleural and peritoneal tumour growth in rats.

**DOI:** 10.1038/bjc.1974.165

**Published:** 1974-08

**Authors:** M. V. Pimm


**B.C.G. TREATMENT OF PLEURAL AND PERITONEAL TUMOUR GROWTH IN RATS.** M. V. PIMM. Cancer Research Campaign Laboratories, University of Nottingham.

Growth of intrapleurally injected cells of immunogenic 3-methylcholanthrene induced rat sarcomata was suppressed by intrapleural injection of viable or radiation sterilized B.C.G. organisms. As little as 10 $\mu g$ of B.C.G. was effective and treatment was successful even if given several days after tumour challenge. B.C.G. administered intravenously or subcutaneously was without influence on pleural tumour growth. Similarly, peritoneal growth of these sarcomata was suppressed only by the intraperitoneal injections of B.C.G. organisms.

In contrast, pleural or peritoneal growths from cells of other rat tumours, which lacked significant immunogenicity, were not controlled by this type of B.C.G. treatment. Nevertheless, these studies indicate the potential of B.C.G. (contact) immunotherapy for treatment of thoracic and peritoneal tumour deposits, which might perhaps have a clinical application, for example in the treatment of malignant mesothelioma of the pleura and peritoneum.